# The Role of Photo-Cycles in the Modulation of Growth and Biochemical Profile of Microalgae: Part I—Food Interest Compounds

**DOI:** 10.3390/life12030462

**Published:** 2022-03-21

**Authors:** Rafaela Basso Sartori, Raquel Guidetti Vendruscolo, Stephanie Reis Ribeiro, Valcenir Júnior Mendes Furlan, Roger Wagner, Leila Queiroz Zepka, Eduardo Jacob-Lopes

**Affiliations:** 1 Bioprocess Intensification Group, Federal University of Santa Maria (UFSM), Santa Maria 97105-900, RS, Brazil; rafabasso.sartori@gmail.com (R.B.S.); raquelgvendruscolo@gmail.com (R.G.V.); stephanieribeiro18@hotmail.com (S.R.R.); rogerwag@gmail.com (R.W.); zepkaleila@yahoo.com.br (L.Q.Z.); 2 Food Science and Technology, Federal University of Pampa (UNIPAMPA), Itaqui 97650-000, RS, Brazil; juniorfurlan@yahoo.com.br

**Keywords:** *Scenedesmus obliquus*, photosynthesis, photoperiod, lipids, fatty acids, proteins, amino acids, sterols

## Abstract

The objective of this work was to evaluate the effect of different photo-cycles on the growth and biochemical profile of *Scenedesmus obliquus* CPCC05, focusing on food interest compounds. The photo-cycle conditions were separated into three groups: long-term photo-cycles (24:0, 22:2, 20:4, 18:6, 12:12, and 10:14 (h:h)), frequency photo-cycles (2, 4, 8, 12, 24, and 48 times per day (t/d)), and short photo-cycles (0.91:0.09, 0.83:0.17, 0.75:0.25, and 0.50:0.50 (s:s)) of light:dark, respectively. The results showed these microalgae can store enough energy to support cell growth for continuous periods of up to 2 h in the dark, without affecting the productivity of the process. This 2 h, when divided into 2 cycles per day (2 t/d), showed the best growth condition (3700 mg L^−1^), generation time (14.40 h), and maximum biomass productivity (21.43 mg L h^−1^). This photo-cycle of 2 t/d was also the best condition for the production of total sterols. However, the values of polyunsaturated fatty acids, lipid content, and amino acids obtained higher yields in the short photo-cycle of 0.75:0.25. Thus, the modulation of light cycles becomes an important tool for boosting and directing the production of target molecules in phototrophic cultures of microalgae.

## 1. Introduction

Over the last few years, changes in eating habits and changes in nutritional requirements have led to considerable changes in food formulation, shifting consumption trends towards natural products with functional properties driving the exploration of alternative food sources [1]. In this context, the biotechnology of phototrophic microorganisms has been extensively developed using microalgae. The biomass of these microalgae can be considered as one of the most promising sources for high-value bioactive products for nutraceutical and pharmaceutical applications, as well as supplements in human and animal nutrition [2].

Among the microalgae species, the genus *Scenedesmus*, belonging to the *Chlorophyceae* class, are very attractive microalgae due to their biological plasticity. *Scenedesmus obliquus*, in particular, is characterized by having high growth rates, being a high source of interesting compounds, and having a valuable biochemical profile [3].

In the state of the art, low volumes and high costs of biomass production impede the commercial competitiveness of microalgae-based products [4]. As most microalgae products are intracellular, their economic viability strongly depends on biomass productivity. In order to use microalgae as a source of product, it is important to study the influence of cultivation conditions as well as the rate of product generation [5]. Research shows that light availability is one of the main factors in microalgae growth and biomass accumulation, due to an increase in the rate of photosynthesis [6,7]. Indeed, in higher plants as well as in green microalgae, antenna pigment molecules bound to light-gathering complexes in the thylakoid membrane help to collect light and transfer energy to the reaction center of photosystems, e.g., PS II. These pigments are responsible for absorbing light and protecting the photosynthetic apparatus against excess light [8,9].

As microalgae growth depends on the amount of light energy offered by intensity and photoperiod, both factors must be taken into account when determining ideal light conditions [10]. In microalgae cultures, exploration of sunlight as a light source is advantageous because it is free and abundant. However, it also has certain disadvantages such as day/night cycles, changing weather conditions, and seasonal changes. In addition, all these factors are site-specific [11]. These luminosity variations can be avoided when applied in cultivation systems controlled through closed photobioreactors [12].

Although many advantages have led to numerous initiatives where artificial lighting is used in photobioreactors, there is still no economic viability in many of the processes of industrial interest. To better estimate and optimize microalgae productivity under different conditions, it is necessary to model the process to obtain useful information about the performance of microalgae farming systems. In this case, the modulation of light cycles can reduce the demand for light energy and, consequently, develop cheaper and more efficient processes [13].

Finally, light conditions such as intensity and photoperiods play irreplaceable roles in the biological metabolic activities of microalgae. Several previous studies reported that, in addition to light intensity, light/dark cycles not only affected the growth of photosynthetic microorganisms but also significantly altered the production of key compounds in microalgae [14,15]. In fact, optimal periods of light allow cells to grow and can make the most of this energy metabolically when available sufficient amounts of light/dark. For this, it is known that photosynthetic metabolism allows an accumulation of energy during the supply of light, thus ensuring that the process can be continued for some time in the absence of light. In this line of work, the most used cycles are between 24 h:0 h and 12 h:12 h light:dark, and they can be divided into two or more cycles per day or in a proportion of seconds or milliseconds [16]. Thus, knowing the factors that determine the biomass productivity and the biochemical behavior in the profile of microalgae in the face of the different cultivation conditions, it is possible to elucidate more efficient strategies and optimize scaling for productions that cover the current global needs for food.

In this sense, the objective of this work was to evaluate the role of photo-cycles (long-term, frequency, and short photo-cycles) in the growth and biochemical profile of *Scenedesmus obliquus*, focusing on food interest compounds, more specifically in lipids, protein amino acids, and sterols.

## 2. Materials and Methods

### 2.1. Microalgae and Culture Media

Axenic cultures of *Scenedesmus obliquus* (CPCC05) were obtained from the Canadian Phycological Culture Centre. Stock cultures were propagated and maintained in synthetic BG-11 medium, with the following composition (mg L^−1^): K_2_HPO_4_ (3.0), MgSO_4_ (75.0), CaCl_2_·2H_2_O (36.0), ammonium citrate and iron (0.6), Na_2_EDTA (1.0), NaCl (0.72), NaNO_3_ (150.0), citric acid (0.6), Na_2_CO_3_ (15.0), H_3_BO_3_ (2.8), MnCl_2_ 4H_2_O (1.8), ZnSO_4_·7H_2_O (0.22), Na_2_MoO_4_·2H_2_O (0.39), CoSO_4_·6H_2_O (0.04) [17]. The incubation conditions used were 30 °C, photon flux density of 30 μmol m^−2^ s^−1^ and a photoperiod of 12 h. 

### 2.2. Photobioreactor Design

Measurements were made in a bubble column photobioreactor (Tecnal, Piracicaba, Brazil). The system was built in 4 mm thick glass with an internal diameter of 7.5 cm, a height of 75 cm, and a nominal working volume of 2.0 L. The dispersion system for the reactor consisted of a 1.5 cm diameter air diffuser located in the center of the column. The reactor was illuminated with forty-five 0.23 W LED lamps (total consumption of 0.01125 kWh), located in a photoperiod chamber. The CO_2_/air mixture was adjusted to achieve the desired concentration of carbon dioxide in the airstream, through three rotameters that measured the flow rates of carbon dioxide, air, and the mixture of gases, respectively.

### 2.3. Obtaining the Kinetic Data in an Experimental Photobioreactor 

The experiments were carried out in bioreactors operating on batch mode, fed with a 2.0 L synthetic BG-11 medium. The experimental conditions were as follows: initial cell concentration of 100 mg L^−1^, isothermal reactor operating at a temperature of 26 °C, photon flux density of 150 μmol m^−2^ s^−1^, and continuous aeration of 1 VVM (volume of air per of culture per minute) with the injection of air enriched with 15% carbon dioxide. In the experiments with long-term photo-cycles, the evaluated light cycles were (h:h) 24:0, 22:2, 20:4, 18:6, 12:12, and 10:14 (light:dark). In the frequency photo-cycles experiments, the cells were exposed to 22 h of light and 2 h of darkness (optimal condition previously defined). These 2 h were divided into six frequencies: 2, 4, 8, 12, 24, and 48 times per day (t/d). To study the effects of short photo-cycles, four different cycles of (s:s) 0.91:0.09, 0.83:0.17, 0.75:0.25, and 0.50:0.50 (light:dark) were set every one second. Cell concentration was monitored every 24 h during the growth phase of the microorganism. All experiments were conducted until the declining phase. The tests were carried out in duplicate, and the kinetic data referred to the mean of four repetitions.

### 2.4. Total Lipids and Fatty Acids Profile

The total lipids were extracted with methanol: chloroform mixture according to the method described by Vendruscolo et al. [18]. Part of the organic fraction containing the lipids was placed in a beaker previously tared, and the content of total lipids was determined by gravimetry on an analytical balance with 0.01 mg minimum display (AUW220D, Shimadzu, Kyoto, Japan) after solvent evaporation in air circulation oven (B5AFD, DeLeo, Porto Alegre, Brazil) at 105 °C. The rest of the organic fraction was derivatized with 1 mL of methanolic KOH (0.4 M) followed by 3 mL of methanolic H_2_SO_4_ (1 M); after each addition, the samples were kept for 10 min in a heating block (CE-350/25, Cienlab, São Paulo, Brazil) at 100 °C (Hartman and Lago, 1973) to obtain fatty acid methyl esters (FAME). The FAME was recovered hexane and analyzed using a Varian 3600 gas chromatograph equipped with a flame ionization detector (GC-FID) (USA) and Varian 8200 autosampler (USA). A volume of 1 μL was injected in splitless mode (0.8 min split-valve off, then split 20:1), and the FAME was separated in an HP-88 capillary column (Agilent Technologies, Santa Clara, CA, USA) (100 m × 0.25 mm × 0.20 μm). The column temperature was held at 50 °C for 1 min; then, it was increased to 185 °C at a rate of 15 °C min^−1^, followed by an increase at 0.5 °C min^−1^ to 190 °C and finally at a rate of 15 °C min^−1^ until reaching 230 °C, remaining for 10 min. The injector and detector temperature was 250 °C. FAME was identified by comparison with authentic standard FAME Mix 37 (P/N 47885-U, Supelco, Bellefonte, PA, USA). Results were expressed as a percentage of the total area of the chromatograms, considering the correction factors of the flame ionization detector and conversion of the ester to acid [19]. The determinations were carried out in triplicate.

### 2.5. Protein Amino Acids Profile 

To determine the protein amino acid profile, 100 mg of biomass were subjected to acid hydrolysis with 6 M hydrochloric acid (HCl) at 110 °C (Heating Block CE-350/25, Cienlab, São Paulo, Brazil) for 24 h under vacuum [20]. The hydrolysates (100 µL) and the norleucine (P/N N1398, Supelco, Bellefonte, PA, USA) internal standard (100 µL, at 10 µg mL^−1^) were evaporated at 60 °C in a heating block (CE-350/25, Cienlab, São Paulo, Brazil) with nitrogen flow. To the residue were added 50 µL of acetonitrile and 50 µL of MTBSTFA (*N*-Methyl-*N*-tert-butyldimethylsilyltrifluoroacetamide) and maintained for another 120 min at 100 °C to occur the silylation reaction. After derivatization, 1 µL of the samples was injected in splitless mode (1 min split-valve off, then split 10:1) in a gas chromatograph coupled to a mass spectrometer (GC-MS) (Shimadzu Corporation QP-2010 Plus, Kyoto, Japan). The injector was at 285 °C. Helium was used as a carrier gas with a constant linear velocity rate of 40.0 cm s^−1^. The compounds were separated in NST-5MS capillary column (NST, Brazil; 30 m × 0.25 mm × 0.25 µm). The initial column temperature was 100 °C for 1 min, followed by an increase to 220 °C at a rate of 20 °C/min, then to 250 °C at 5 °C/min. Then the temperature reached 260 °C with an increase of 2 °C/min remaining for 1 min, after which it reached 265 °C at 2 °C/min. Then, it was raised to 285 °C at 5 °C/min, remaining in isotherm for 1 min. Finally, it reached 300 °C at a rate of 15 °C/min where it was held for 2 min. The analytes were positively identified by comparing the retention times of the authentic standards of 21 amino acids (P/N LAA21, Supelco, Bellefonte, PA, USA) and the mass spectra found in the spectral library of the National Institute of Standards and Technology (NIST 05s). Quantification was performed by external calibration (0.2–10 µg mL^−1^) with areas previously normalized by the internal standard. The determinations were carried out in triplicate. 

### 2.6. Sterols Profile

The sterols extraction from biomass was performed by a direct saponification method according to [21], with some adaptations. Briefly, 50 mg of dry biomass was weighed. After 1 mL of ethanolic KOH solution (10% *w*/*v*) was added and submitted to a horizontal (150 rpm, 40 °C per 6 min). Then, 1 mL of salt-saturated solution (36%) was added, and 1 mL of chloroform was added twice in order to finish the saponification procedure. The organic fraction was dried under nitrogen, and sterol compounds were suspended in 100 µL of 3:2 (hexane: isopropanol) solution and submitted to gas chromatography analysis, in GC-FID as previously described in item 2.4. The sterols were quantified by using seven-point analytical curves, and the curves were as follows: 20–700 μg mL^−1^ for tocopherol, stigmasterol, and β-sitosterol and 1.25–50 μg mL^−1^ for squalene. The linear range was defined according to the sterols concentration found in the samples. To the linearity study, a linear regression equation was used, and the linear correlation coefficient of the calibration curve was determined. The limit of detection was estimated according to the concentration of the compound at a signal-to-noise ratio of 3. The identification of the compounds was performed by using gas chromatography coupled to a mass spectrometer (GC/MS), Shimadzu QP-2010 Plus (Tokyo, Japan), at the same chromatographic conditions as those described for GC-FID, except the carrier gas that was used helium. The GC/MS interface and ion source were held at 280 °C. The MS ionization source was operated in electron impact (EI) at +70 eV and the single quadrupole mass analyzer in scan mode (35–450 *m*/*z*). The compounds above cited were positively identified by a comparison of the retention time and mass spectra obtained experimentally and authentic standard. The other sterols were tentatively identified by mass spectra experimental comparison with those obtained from NIST 05 library (NIST 05, Gaithersburg, MD, USA).

### 2.7. Statistical Analysis

The Statistica 10.0 software (StatSoft, Tulsa, OK, USA) was used to test differences between treatments by analysis of variance (one-way ANOVA) and Tukey’s test. In addition, all data were submitted to Principal Component Analysis (PCA) using Pirouette 3.11 software (Infometrix Co., Bothell, WA, USA).

## 3. Results and Discussion

**Figure 1:**
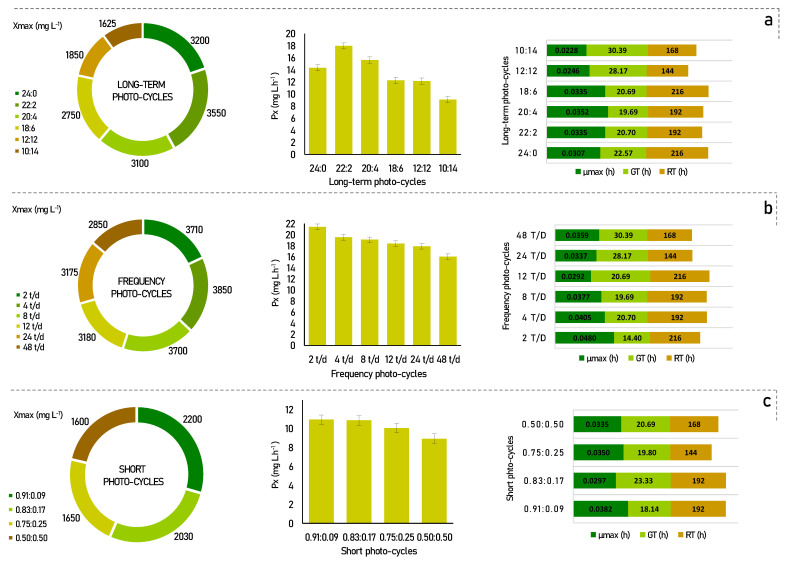
Kinetic parameters for *Scenedesmus obliquus* in different photo-cycles: (**a**) long-term photo-cycles; (**b**) frequency photo-cycles; (**c**) short photo-cycles. X_max_-maximum cell growth; Px-biomass productivity; µ_max_-maximum specific growth rate; GT-generation time; RT-residence time.

Microalgae are selective organisms, and the influence of the cultivation conditions to which they are exposed can provide a potential increase in biomass growth and productivity, as well as in the production of metabolites of interest [22]. In view of this, to study the effect that photo-cycles exert on *Senedesmus obliquus*, cultivation experiments were carried out, and their results are shown in Figure 1.

According to Figure 1a, for the long-term photo-cycles, the maximum biomass productivity was found to be 22:2 (17.97 mg L h^−^^1^), followed by 20:4 (15.63 mg L h^−1^), and 24:0 (14.35 mg L h^−1^). In comparison, the other photo-cycles showed lower cell productivity, with, 12.27 mg L h^−1^, 12.15 mg L h^−1^, and 9.08 mg L h^−1^, for photo-cycles of 18:6, 12:12, and 10:14, respectively.

It is recognized that more light provides more energy for the development of microalgae, but their development can be reversibly inhibited when this amount of light becomes too high. In previous studies, similar results have shown that increased light availability increased microalgae productivity up to a certain interval [23,24]. This was also stated by Liu et al. [14], in which he reports that experiments with full light were not favorable, but that an adequate light/dark cycle ratio was better. Appropriate stimulation of the dark can increase the growth of the photosynthetic microorganisms, temporarily altering the intracellular enzymatic reactions. However, a condition that is too long and too dark can reduce the photosynthetic system and inhibit cell growth due to insufficient ATP, and this can be seen in the low productivity and cell growth of the 10:14 photo-cycle, as shown in Figure 1a.

Although some differences have been noted in the results cycles light/dark of long-term considered by Liu et al. [14], the best performance of the process for *Chlorella vulgaris* was also observed in photo-cycles supported in periods of low darkness (maximum of 4 h). Jacob-Lopes et al. [13] also studied different photoperiods in the cultivation of the cyanobacterium *Aphanotece microscopica Nägelli* and found that this species managed to maintain high productivity with the same 22:2 h light/dark photo-cycles. Thus, these results suggest that this is the best light/dark condition in long-term photo-cycles for *Scenedesmus obliquus* CPCC05, in which enough energy can be stored to support cell growth, without affecting the rate of photosynthetic metabolism. Therefore, as a strategy to improve the performance of the process, the following step presents the effect of the light/dark 22:2 photo-cycle, divided into different frequencies over the period.

Several authors have already reported that the frequency of the light/dark cycle had great effects on the growth of microalgae, in addition to being a good strategy to improve the performance of the process [25,26]. As shown in Figure 1b, an improvement can be seen in most process parameters for frequency photo-cycles. This behavior was observed up to 12 t/d, where the best cell productivities were found when compared to the long-term photo-cycles. Furthermore, there is a linear reduction behavior in this kinetic parameter for all photo-cycles. Cell productivity values were 21.43, 19.53, 19.10, 18.40, 17.90, and 16.02 mg L.h^−1^ for photo-cycles of 2 t/d, 4 t/d, 8 t/d, 12 t/d, 24 t/d, and 48 t/d, respectively. 

If we compare the frequency cycles of 2, 4, 8, and 12 times a day with constant lighting, there was a gain of 2.39% to 16.15% in biomass productivity. This behavior is probably associated with the photoinhibition phenomenon under the condition of constant lighting. This occurs when photosynthetic organisms are exposed to strong light, which results in the inhibition of photosystem II activity [27]. Moreover, according to Liao et al. [28], process performance is benefited with higher frequencies of light/dark cycles per day. However, this was not confirmed in our results, since for the photo-cycle of 2 t/d, the cell productivity achieved a performance of more than 33% when compared to the higher cycle frequencies, such as 48 t/d. In this case, dividing the light/dark cycles by 2 t/d can benefit the optimal distribution of energy in the photosynthetic apparatus [29].

In specific situations, the increase in photosynthetic efficiency must be considered when evaluating the fluctuations effect of the light/dark cycle. These fluctuations can be explained by the fact that light energy can be used more efficiently if it is delivered in small pulses throughout the process compared to a constant flow. These small pulses can lead to changes in the oxidation/reduction state that directly influence the antenna of the photosynthetic apparatus [29].

The kinetic parameters in short photo-cycles were determined under 4 different light regimes, as shown in Figure 1c. Under the applied regimes, the best conditions were found for 0.91:0.09, 0.83:0.17, and 0.75:0.25 with cell productivity of 10.94, 10.86, and 10.05 mg L h^−^^1^, respectively. The photoperiod of 50:50 showed less efficient results than the others (8.93 mg L h^−1^). These results show that under short light/dark cycles, the rate of cell productivity decreases proportionally to the fraction of light used during the period. These data suggest that the more the microalgae cells are exposed to light, the more light energy is stored for use in dark periods of a few hours [15]. This is clear evidence that considerable light integration occurs when the cycle time is short enough.

Thus, when compared to the long-term experiments, the short photo-cycles achieved almost 38% less cell productivity when analyzing their best rates. In fact, the stress resulting from very short periods can become excessive and cause an increase in cell mortality and a decrease in cell productivity, as discussed, for example, by Chiarini [15]. In our results, the preference for low darkness cycles was evident, and consequently, dividing these periods up to a certain limit can positively impact the microalgae culture. The 2 t/d photo-cycle showed the best rates of kinetic parameters, as observed in Figure 1.

### 3.1. Analysis of the Biochemical Profile 

#### 3.1.1. Total Lipids and Fatty Acids

Lipid components play key roles in membrane structure and metabolic processes in microalgae. Table 1 presents total lipid content and fatty acid (FA) composition obtained from the microalgae *Scenedesmus obliquus* cultivated with different photo-cycles.

When the results in Table 1 and Figure 1a are compared, lipid content and cell productivity were inversely proportional in the long-term photo-cycles. The higher lipid content obtained with the cultivation in a photo-cycle of 10:14 (16.60%) and 18:6 (16.28%) can be attributed to the storage of chemical energy in the form of oil, as neutral lipids or triglycerides, by microalgae under stress conditions [30]. The same behavior was observed by Vendrusculo et al. [18] when they evaluated the influence of 24:0 and 12:12 photoperiods for *Scenedesmus obliquus*, in which higher light incidence provided greater growth with less lipid accumulation. This behavior is believed to occur to protect cells from photochemical damage.

For frequency photo-cycles, the highest lipid content occurred 8 and 4 times a day, reaching 16.87% and 15.8%, respectively. However, the condition that presented the highest lipid content of all photo-cycles was the short photo-cycle of 0.75:0.25, reaching a total of 23.26%, followed by the photo-cycle of 0.83:0.17 (19.69%). These results may be related to the fact that small pulses of light stimulate the synthesis of fatty acids through the fluidity of membranes under adverse conditions since higher lipid yields are not obtained under ideal growth conditions [31].

According to Table 1, twelve fatty acids were detected in the *Scenedesmus obliquus* for all photo-cycles and, as expected, there was variability in the AG composition for different light conditions. The cultures under higher light incidence had higher concentrations of saturated and monounsaturated fatty acids when compared to polyunsaturated fatty acids, obtained under atypical conditions for their ideal growth, for example, cycles with longer dark times, frequency, and pulse.

As expected, the 24:0, 22:2, and 20:4 h photo-cycles and 2 t/d the saturated fatty acid content was around 50–55%. These values are inversely proportional to the 10:14 and short photo-cycles. In these cycles, the variability of PUFAS occurred between 46–53%. This can possibly be explained by the fact that as the unsaturations are prone to oxidation (e.g., light, temperature, oxygen), the polyunsaturated fatty acids are the first to be degraded in photoperiods with a higher incidence of light, in this case, cycles of 24:0 and 22:2 [32]. Compared with cultures under constant lighting, higher PUFA concentrations in cultures with the 12:12 photoperiod was also observed for *Scenedesmus* sp. and *Chlorella minutissima* [33,34].

The dominant fatty acids were palmitic acid (C16:0), alpha-linolenic acid (C18:3n3), and oleic acid (C18:1n9). In general, the lipid profile found in this study is commonly found in different members of *Chlorophyceae*, such as *Botryococcus* sp., *Dunaliella salina*, *Chlorella* sp., and *Chlorococcum* sp. [35,36].

#### 3.1.2. Protein Amino Acid

**Figure 2:**
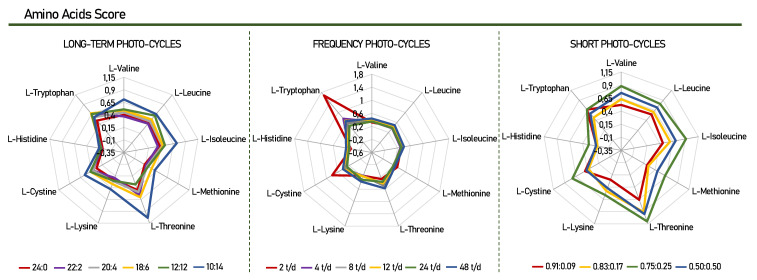
Chemical score of the essential amino acids found *Scenedesmus obliquus*. Amino Acid Score—mg of amino acid per g test protein/mg of the same amino acid per g reference protein [37].

Twenty-two amino acids (AA) were detected in *Scenedesmus obliquus* microalgae for all groups of photo-cycles, as described in Table 2. The essential AA identified and quantified were valine, leucine, isoleucine, methionine, threonine, phenylalanine, lysine, histidine, and tryptophan. In addition, thirteen non-essential AA were also found (alanine, glycine, proline, serine, aspartic acid, hydroxyproline, cysteine, glutamic acid, asparagine, glutamine, cystine, arginine, and tyrosine), with the exception of glutamine in the photo-cycles of 2, 4, 24, and 48 t/d. Figure 2 presents chemical score of the amino acids found in *Scenedesmus obliquus* for this study.

According to Table 2, the short photo-cycle of 0.75:0.25 was the one that most stimulated the production of essential amino acids, with a productivity of 213 mg g^−1^. Periods of 10:14 and 48 t/d of frequency also influenced the production of these amino acids, with 167.50 and 110.30 mg g^−1^, respectively. When comparing these values with the use of continuous light (24:0), there was a gain of more than 20%. Prates et al. [38] also found the same behavior for *Spirulina* sp., which obtained a gain of 18% when compared the photo-cycles 12:12 and 24:00, confirming that in periods of darkness, the productivity of essential amino acids obtained the best condition. 

After analyzing the number of essential amino acids in the culture, the highest production of leucine (leu) and valine (val) was highlighted, in that order, for all photo-cycles. The assessment of essential amino acids is vital when evaluating the nutritional value that a protein product can arrange. Various methods have been developed and used to assess the quality of the protein of a given product, for example, the composition of amino acids (Table 2) and the score of these amino acids in comparison with the requirement standard proposed by the FAO/WHO (Figure 2) [39].

Through the chemical scores, it was possible to identify the nutritional value and determine the order of the limiting amino acids in this study. For example, a chemical score below 1.00 means that the protein studied has at least one essential amino acid in insufficient numbers [40]. The results presented in Figure 2 show that for *Scenedesmus obliquus*, histidine is considered the first limiting amino acid, as it presented the lowest chemical score value when compared, for all photo-cycles. Histidine has been reported in other studies to be the limiting amino acid for *Scenedesmus obliquus*, *Arthrospira platensis*, and *Botryococcus braunii* [41,42,43]. In contrast, these microalgae proved to be a good source of tryptophan and threonine with scores of 1.68 and 1.10, for cycles of 2 t/d, 0.75:0.25, respectively. Considering that tryptophan and threonine are nutritionally important, the variation of different photo-cycles presented in this study can change the profile and composition of essential amino acids, according to each specific need. This makes *Scenedemsus obliquus* capable of producing high-quality protein when compared to the FAO/WHO reference standard.

As for the productivity of non-essential amino acids, the best results were also for photo-cycles of 0.75:0.25 (410.60 mg g^−1^) and 10:14 (316.90 mg g^−1^). Among all compounds in this group, arginine was one of the amino acids found in higher concentrations in both photo-cycles, reaching a productivity of 94.40 mg g^−1^ for 12 t/d. Arginine is proteinogenic and acts as an essential metabolite in many cellular and developmental processes which can induce microalgae tolerance to abiotic stress. However, to date, few studies have focused on the role of this amino acid in algae stress tolerance [44].

The analysis of Table 2 also identified the non-essential compounds of alanine, proline, aspartic acid, and glutamic acid as the main constituents of the protein fraction of *Scenedesmus obliquus.* The pathways for the synthesis of alanine have physiological significance and result in cellular osmoregulation. Furthermore, it was demonstrated that alanine accumulation is a general phenomenon exposed to a variety of factors and is universally one of the first signs of stress expressed by cells [45]. These results corroborate those found in this study since alanine production was higher in dark photo-cycles (18:6, 12:12, and 10:14), higher frequency per day (48 t/d), and in the short photo-cycle (0.75:0.25). 

In conclusion, the total amino acid data obtained in all photo-cycles may be related to the lower microalgal growth and productivity, observed in Figure 1, so that the amino acids remain free in the intracellular medium. In this case, the amino acid is used to a lesser extent by the metabolic pathways for polymeric formation, which can end up accumulating in the intracellular environment, resulting in the high accumulation of these components in cells [18].

#### 3.1.3. Total Sterols 

Sterols are essential components of the membrane, regulating membrane fluidity, and permeability in a way that is essential for microalgae survival [46,47]. Table 3 presents the sterol content identified for *Scenedesmus obliquus.*

The total sum of sterols found in all photo-cycles is better visualized in Table 3. A great variation is observed between the groups, in which the photo-cycles 18:6, 2 t/d, and 0.83:0.17 were the majority, for the long-term, frequency, and short groups, respectively. In fact, sterol production is positively linked to the fact that an adequate light/dark ratio provides better results. This suggests that specific sterol molecules within microalgae can be positively affected when grown under ideal conditions. In this case, the high sterol content found at 2 t/d (735.27 mg kg^−1^) is closely linked to the high cell productivity found for this same photo-cycle [48].

When the microalgae undergo some type of stress (either by too much or too little light or when there is some variation in the medium), they can undergo rapid downregulation of genes involved in sterol biosynthesis, resulting in an impaired photosynthesis system. In this case, the high number of cycles used in the 48 t/d photo-cycle may have influenced the low accumulation of sterols. This inhibition also resulted in severe deviations of the chloroplast structure, reduced photosynthesis rates, and low-level sterols for *Nannochloropsis oceanic* [49]. The involvement of sterols in the chloroplast is also exemplified in the microalgae *Euglena gracilis*, which did not produce sterols when cultivated in the absence of light [48].

In the present study, five types of sterols were identified (squalene, tocopherol, ergosterol, stigmasterol, and β-sitosterol). However, tocopherol was not found in short photo-cycles and in periods of 24 and 48 t/d. In the long-term, this compound is only visualized in 22:2. Likewise, β-sitosterol is not observed in 48 t/d and in long-term foto-cycles, with the exception of 18:6.

According to Table 3, the main compound identified as stigmasterol, followed by ergosterol, with the productivity of 450.16 and 142.52 mg kg^−1^ for 2 t/d, respectively. These compounds were also identified in all photo-cycles, as well as squalene. The productivity of these sterols is described as a result of the stimulation of photosynthesis by the MEP pathway at longer periods of light. The MEP pathway is hypothetically related to photosynthesis since it is bound to the chloroplast [50].

Varied abiotic effects have been reported to improve phytosterol production in some microalgae species. For example, sterol levels increased with a higher light period, mainly in eukaryotic microalgae [51,52]. In fact, eukaryotic microalgae, such as *Scenedesmus obliquus*, are reported in the literature as the most prominent strains for the production of sterols and are important to enable the cellular permeability of the membrane and maintain structural protection, highlighting in the environmental adaptation [21,53]. 

In summary, *Scenedesmus obliquus* proved to be a great ally in the production of sterols, especially when cultivated under ideal growth conditions and productivity. Furthermore, phytosterols such as ergosterol and stigmasterol are considered a trademark in microalgae, which can also be confirmed for *Scenedesmus obliquus*, as they were found in significant amounts for all photo-cycles used. Phytosterols are similar to cholesterol and despite being smaller in structure, they are important precursors in intracellular traffic and metabolic actions. In addition, experimental studies have shown that squalene leads to an increase in microalgal performance. This can be understood by the essential role of squalene in protecting the biomembrane of cells against oxidative stress, providing a more efficient metabolism and contributing to cell maintenance [49]. Finally, these findings provide new insights into improving sterol production in different photo-cycles for manipulation of a particular biochemical profile of interest.

### 3.2. Biochemical Pathways of Food Interest Compounds

**Figure 3:**
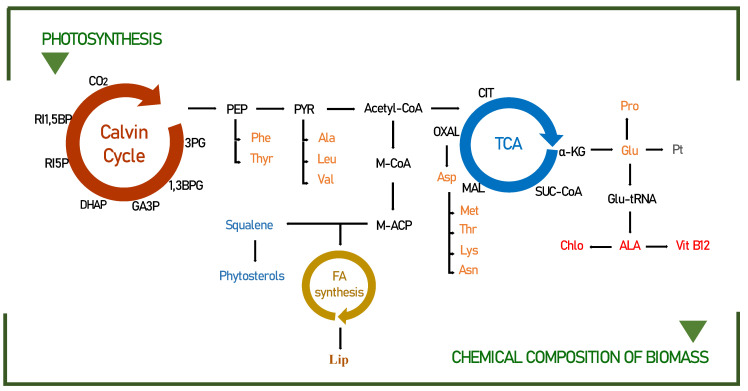
Pathways leading to the biochemical compounds identified in this study for *Scenedemus obliquus*. Modified from the following [18,55,56]. 3PG—3-phosphoglycerate; 1,3BPG—1,3-biphosphoglycerate; GA3P—glyceraldehyde-3-phosphate; DHAP—dihydroxyacetone phosphate; RI5P—ribulose-5-phosphate; RI1,5BP—ribulose-1,5-bisphosphate; PEP—phosphoenolpyruvate; PYR—pyruvate; Acetyl-CoA—acetyl coenzyme A; Phe—phenylalanine; Thyr—tyrosine; Ala—alanine; Leu—leucine; Val—valine; M-CoA—malonyl coenzyme A; M-ACP—malonyl–acyl carrier protein; FA—fatty acids; Lip—lipids; CIT—citrate; α-KG—α-ketoglutarate; SUC—succinate; MAL—malate; OXAL—oxaloacetate; Asn—asparagine; Asp—aspartic acid; Lys—lysine; Thr—threonine; Met—methionine; Glu—glutamic acid; Glu-tRNA—glutamate-tRNA; Pro—proline; ALA—5-aminolevulinate; Vit B12—Vitamin B12; Chlo—chlorophyll; Pt—protein.

Under specific growth conditions, microalgae assimilate a certain amount of fatty acids, amino acids, and sterols [54]. The amount and quality of light imposed play an essential role in determining the biofixation of CO_2_, which consequently leads to an accumulation of significant amounts of adenosine triphosphate (ATP), nicotinamide adenine dinucleotide hydrogen phosphate (NADPH), and glyceraldehyde-3-phosphate (3GP), indispensable for the formation of these compounds (Figure 3).

By modifying the duration of the light/dark cycles, it is possible to change the biochemical composition of microalgae. For example, the conversions of phosphoenolpyruvate to pyruvate and 3-phosphoglycerate to glycerol 3-phosphate are candidate limiting steps for lipid accumulation during light/dark cycling [57]. The accumulation of substrates, including ribulose 5-phosphate, during dark periods, can be explained by the close relationship of increased biomass yield with improved CO_2_ fixation, related to the metabolic profile, to carbohydrate synthesis and mainly to the lipid pathway (phosphoenolpyruvate; PEP, pyruvate, and acetyl-CoA), as detailed in Figure 3.

To fully demonstrate the potential of the metabolic mechanisms, the lipid production, and fatty acid profile must be determined by directly comparing the different light/dark periods, detailed in Table 1. It has been suggested that at high light periods (in this case photo-cycles between 24:0 and 20:4), microalgae cells use excess energy to produce storage lipids, mainly SFA and MUFA, which are generally involved in the formation of thylakoid membranes [58]. However, when exposure to dark cycles increased from 4 h to 14 h, an obvious drop (over 20%) could be found in the amount of saturated fatty acids. This can be attributed to the influence of photooxidation caused by excessive light time leading to excessive accumulation of dissolved oxygen. Therefore, the hydroxyl radicals produced in this process can promote the transformation of saturated fatty acids into unsaturated fatty acids, especially observed in C16:0 and C18:3n3 [59].

The protein amino acid content of microalgae varies significantly between species and strains [60]. Particularly, in *Scenedesmus obliquus*, the variation in amino acids was visually affected by photo-cycles, as already shown in Table 2.

In the normal metabolic pathway, amino acids are synthesized from 3-PGA, phosphoenolpyruvate, pyruvate, α-ketoglutarate, and oxaloacetate (Figure 3). However, in recent years, several lines of evidence have suggested that amino acids are not only used for the synthesis of storage proteins but also, upon energy demand, their products can be produced in the TCA cycle to generate energy [61,62]. For example, in prolonged darkness, the amino acid asn accumulates, and its role as an energy donor occurs through the conversion of Asn to Asp, which under conditions of energy shortage synthesizes the amino acids lys, thr, and met through the TCA cycle. This can be confirmed in our studies, since the productivity of the amino acids lysine, threonine, and methionine practically doubled in longer periods of darkness (10:14) when compared to the continuous light photo-cycle (24:0).

The productivity of sterols in microalgae can be easily manipulated to increase their concentration through different photo-cycles. Much has been reported that the increase in sterols is closely linked with the best growth condition, in this case, seen in the photo-cycle of 2 t/d. In fact, the biosynthesis of some microalgae shows that the production of sterols occurs through the MEP pathway, as building blocks of IPP and DMAPP from Acetyl-CoA [53]. For this reason, hypothetically related to photosynthesis, the explanation for the higher sterol intensities found for *Scenedesmus obliquus* in this study is obtained. In addition, the effects of light interaction on sterol content may be related to increased carbon fixation, which also allows for greater sterol synthesis [63].

When investigating the relationship between sterol biosynthesis in a given light cycle, we observed that at 22:2, the amount of total sterol obtained smaller proportions than those found at 2 t/d. This suggests a direct correlation among light stress and sterol metabolism. In summary, in response to high light stress, sterol biosynthesis as a whole is suppressed, while modification to optimal light cycles has a high concentration [49]. In this case, total sterols increased from 60.13 to 735.27 mg kg^−1^, when this same proportion of light was fractionated into 2 cycles per day. Intense light is known to cause biochemical damage to the photosynthetic apparatus in microalgae [50].

### 3.3. Exploratory Multivariate Analysis

**Figure 4:**
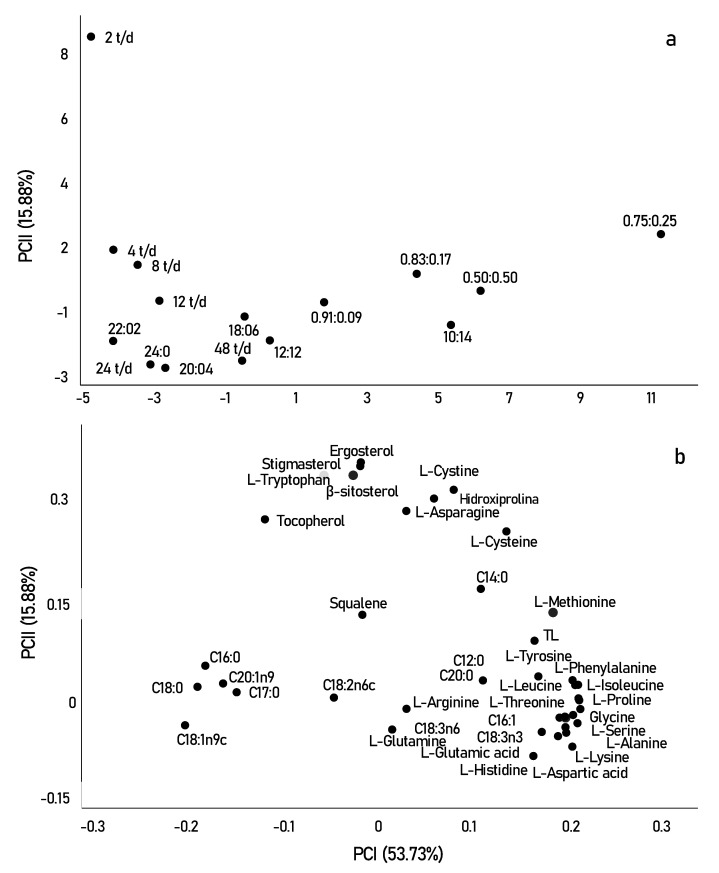
Graphs of scores (samples) (**a**) and loadings (variables) (**b**) showing principal components 1 (PCI) and 2 (PCII) of *Scenedesmus obliquus* cultivated with different photo-cycles.

Principal component analysis (PCA) was used to better visualize the effect of different photo-cycles on the biochemical composition of *Scenedesmus obliquus*. The two main components explained 69.61% of the overall variance of the results obtained. Figure 4 shows the scores (samples) and loads (compounds) of principal components 1 and 2, with 53.73% and 15.88%, respectively. Based on these two PCs, the samples furthest from the origin are presented as the most relevant.

In Figure 4a,b, the samples were clearly separated into three groups (fatty acids, amino acids, and sterols). Initially, in terms of fatty acid contents, the samples grown with the highest light incidence were mainly correlated with saturated fatty acids, such as (C16:0) and (C18:0). In contrast, longer periods of darkness or shorter periods may have directly affected the production of unsaturated compounds such as linoleic acid (C18:2n6) and alpha and beta-linolenic acid (C18:3n6 and C18:3n3).

In fact, in pulsed light experiments, cells are able to avoid photodamage and use the energy from the pulses to improve photon absorption by the photosystem. As described by Maltsev et al. [6] the active functioning of the photosystem, when caused by an active change in light supply, is accompanied by an increase in the desaturation of omega-3 and 6 fatty acids. The desaturation of these fatty acids can be considered as one of the rapid adaptive responses to light pulse changes and may be associated with the “transition states” of photosystems. This implies that short photo-cycles form a good condition for the cultivation of *Scenedesmus obliquus* in pharmaceutical and food industries since beneficial effects on human health have been related to the n-3 LC-PUFA diet, mainly on lipid metabolism [64]. 

In addition to light limitation, the alternation of cycles alters metabolism, through the TCA cycle, resulting in an increase in the storage content of amino acids and stress-related compounds, including the amino acids proline, lysine, and tyrosine, observed for short photo-cycles, in the lower right quadrant [61]. However, when looking at the upper quadrant, we can say that certain amino acids correlate better with longer periods of light. For example, the amino acid tryptophan is strongly related to tryptamine which is a reference indicator of oxidative stress occurring at very high amounts of light [65]. Tryptophan is a nutritionally important essential amino acid, as it is the necessary precursor for the formation of melatonin, a protein that modulates circadian cycles and reduces oxidative stress in most living organisms [66]. The production of melatonin in photosynthetic species occurs most of the time during the day, that is, in longer periods of luminosity. This can be explained by the better results of tryptophan in photo-cycles of 2 t/d, observed in Figure 4.

In summary, we can say that sterols were the most relevant compounds found in this study, as most are significantly separated from the other compounds observed in the two PCs. This result may be strongly related to the high growth and cellular productivity of microalgae when exposed to a frequency of 2 t/d. The frequency of the 2 t/d was mainly correlated with the highest concentrations of stigmasterol, ergosterol, β-sitosterol, and tocopherol. Squalene, however, moved in the opposite direction, as it was correlated and obtained in higher concentrations in photo-cycles of 0.83:0.17, photo-cycle that showed low cell growth and productivity. Squalene is the first compound obtained in the synthesis of sterols, and in most cases, it is inversely correlated with photosynthetic performance, showing that sterols can present a bidirectional response to different photo-cycles [48]. The sterol profiles in microalgae are of great interest in research directed to food importance. While the introduction of these compounds is not a direct replacement for medication for hypercholesterolemia, they may help lower or control cholesterol levels, in addition to having anti-inflammatory, neuromodulatory, and neuroprotective activities. Furthermore, ergosterol is the precursor of vitamin D_2_, which can be converted to vitamin D, while squalene has antioxidant and anticancer activities [50].

With PCA analysis, it is possible to verify the compounds formed under different cultivation conditions (Figure 4a,b). The results suggest that, in certain photo-cycles, the production of certain components of nutritional importance is related either to the best photosynthetic rates obtained in cultivation or, when it is a clear response, to oxidative stress caused by metabolism. Finally, our study made it possible not only to verify the total productivity of lipids, proteins, and sterols but also to distinguish the profile of their components in different photo-cycles. In addition, it also helped to elucidate the biochemical pathway in the production of these compounds. It appears that, for each photo-cycle, the productivity and profile of specific components of *Scenedesmus obliquus* are, at least metabolically, quite distinct.

## 4. Conclusions

The process parameters were significantly influenced by the photo-cycles. The condition that showed the best results was the photo-cycle of 22:2 h divided into 2 cycles per day, with cell growth of 3700 mg L^−1^ and biomass productivity of 21.43 mg L h^−1^.

The modulation of photo-cycles in long-term, frequency, and short photo-cycles also proved to be an effective strategy to improve cultivation conditions and, thus, enable the use of artificial lighting for the production of interesting biochemical components from lipid profiles, protein amino acids, and sterol content. A combined total of twelve fatty acids were identified for the different photo-cycles, while twenty-two amino acids were found in total. For the sterol group, five compounds were identified in the study.

Notably, a significant increase in lipid content and PUFAS concentration was demonstrated under darker conditions (10:14) and mainly under pulsed lighting conditions (0.75:0.25), reaching a lipid content of 23.26%. These photo-cycles also played a significant increase in essential and non-essential amino acids, with the productivity of 213 and 410.60 mg g^−1^, respectively. In darker conditions and pulsed light cycles, the accumulation of certain compounds may be related to imposed stress conditions and/or to protect the photosynthetic apparatus against oxidative damage to exposure to many periods of light.

The Amino Acid Score was presented as a way to demonstrate that *Scenedesmus obliquus* can play a significant role with high nutritional value (1.68), mainly in the production of tryptophan in a 2 t/d photo-cycle. Furthermore, total sterol levels (735.27 mg kg^−1^) were correlated with photo-cycles of periods of high luminosity and greater growth and productivity of biomass cells, results closely linked to better photosynthesis conditions in photoperiods with a frequency of 2 t/d.

Finally, with these results, it was possible to verify the compounds formed and identify the different metabolic transformations that can occur in microalgae cultures, in order to select the most favorable conditions for the production of precursors or bioproducts of food interest. Compounds such as omega-3 and 6, essential amino acids, and phytosterols and squalene, found in the different photo-cycles of *Scenedesmus obliquus*, have great biotechnological potential for commercialization, as they have already been described as excellent nutritional sources for food. Interestingly, the best conditions found in the same photo-cycle both for the production of total lipids and essential amino acids (0.75:0.25), as for the total sterol content and the highest chemical amino acid score at 2 t/d, may provide a potential for simultaneous co-production of these compounds in order to present better efficiency and cost-effectiveness to be used in industrial food processes.

## Figures and Tables

**Table 1:** Total lipids and fatty acids (%) for *Scenedesmus obliquus* in different photo-cycles.

Compounds	Long-Term Photo-Cycle						Frequency Photo-Cycle						Short Photo-Cycle			
	24:0	22:2	20:4	18:6	12:12	10:14	2 t/d	4 t/d	8 t/d	12 t/d	24 t/d	48 t/d	0.91:0.09	0.83:0.17	0.75:0.25	0.50:0.50
C12:0	0.83	0.43	0.90	1.10	2.42	1.68	0.90	0.82	0.96	1.27	1.13	1.90	2.73	3.03	5.04	0.97
C14:0	0.38	0.27	0.39	0.32	0.56	0.41	0.53	0.44	0.50	0.45	0.44	0.48 ^a^	0.50	0.47	0.61	0.57
C16:0	45.66	38.18	44.71	36.72	39.83	36.87	47.57	43.95	45.86	47.02	44.61	40.41	37.37	32.75	32.87	35.04
C16:1	0.60	0.38	0.43	0.51	0.60	0.73	0.34	0.29	0.30	0.55	0.34	0.83	0.98	1.00	1.17	1.16
C17:0	0.26	0.34	0.26	0.32	0.30	0.27	0.32	0.33	0.27	0.30	0.31	0.32	0.26	0.25	0.23	0.26
C18:0	3.09	5.04	5.06	3.36	2.75	1.67	4.52	4.73	5.55	3.75	4.33	3.47	2.10	1.74	1.48	1.82
C18:1n9c	16.10	18.45	18.86	14.23	13.37	11.45	16.39	19.50	20.62	16.19	18.30	14.58	11.25	11.03	5.80	6.52
C18:2n6c	11.59	15.90	11.93	12.54	9.57	12.23	13.24	13.81	11.73	14.32	13.29	16.12	13.67	16.54	11.77	10.47
C20:0S	0.21	0.25	0.22	0.22	0.25	0.24	0.20	0.22	0.27	0.16	0.17	0.16	0.28	0.22	0.27	0.31
C18:3n6	0.71	0.72	0.59	0.81	0.87	1.21	0.66	0.66	0.64	0.72	0.74	1.29	0.85	1.17	1.16	1.13
C18:3n3	20.47	19.81	16.44	29.67	29.41	33.16	15.13	15.04	13.10	15.16	16.18	20.25	29.92	31.77	39.56	41.67
C20:1n9	0.10	0.23	0.20	0.21	0.06	0.10	0.20	0.21	0.19	0.12	0.15	0.18	0.07	0.05	0.05	0.07
∑SFA	50.43	44.51	51.54	42.05	46.12	41.13	54.04	50.49	53.41	52.94	51.00	46.74	43.24	38.45	40.51	38.97
∑MUFA	16.80	19.06	19.49	14.94	14.04	12.27	16.93	20.01	21.11	16.86	18.79	15.60	12.31	12.08	7.01	7.75
∑PUFA	32.76	36.43	28.97	43.01	39.85	46.60	29.03	29.50	25.47	30.20	30.21	37.66	44.45	49.47	53.28	52.49
TL	11.89 ^n^	11.41 ^o^	13.74 ^k^	16.28 ^g^	15.30 ^i^	16.60 ^e^	13.33 ^l^	15.81 ^h^	16.47 ^f^	13.29 ^m^	14.82 ^j^	9.30 ^p^	16.66 ^d^	19.69 ^b^	23.26 ^a^	18.21 ^c^

SFA—saturated fatty acids; MUFA—monounsaturated fatty acids; PUFA—polyunsaturated fatty acids; TL—total lipids. Different letters on the same line indicate differences by Tukey’s test (*p* < 0.05).

**Table 2:** Protein amino acids (mg g^−1^) for *Scenedesmus obliquus* in different photo-cycles.

Compounds	Long-Term Photo-Cycle						Frequency Photo-Cycle						Short Photo-Cycle			
	24:0	22:2	20:4	18:6	12:12	10:14	2 t/d	4 t/d	8 t/d	12 t/d	24 t/d	48 t/d	0.91:0.09	0.83:0.17	0.75:0.25	0.50:0.50
L-Alanine	24.10	27.70	27.40	39.30	32.90	42.50	23.30	27.10	26.50	25.40	27.30	32.30	33.80	34.90	45.50	40.50
Glycine	15.40	13.80	14.40	20.40	18.90	27.40	12.30	13.10	13.70	14.60	16.40	22.90	21.60	25.00	33.40	28.90
L-Valine	15.60	14.60	17.60	19.10	19.90	27.50	12.50	13.60	14.70	16.10	13.80	17.00	20.40	24.70	34.40	29.10
L-Leucine	24.40	23.50	27.00	29.80	36.10	38.40	25.90	21.70	22.90	25.20	22.20	29.10	32.30	35.90	47.50	42.50
L-Isoleucine	11.30	10.10	13.00	13.20	14.50	21.70	9.60	8.90	10.00	11.60	9.70	12.10	13.90	18.10	27.20	21.30
L-Proline	26.50	22.50	26.90	33.30	33.70	49.70	22.00	27.90	29.20	31.10	28.80	38.70	41.00	44.90	69.00	54.30
L-Methionine	2.10	2.50	3.10	4.70	3.40	5.80	4.8	3.00	2.70	3.00	2.90	3.70	3.50	4.00	9.90	7.20
L-Serine	13.90	12.80	13.90	16.90	15.00	26.40	9.00	15.30	16.30	14.60	13.50	17.00	16.90	22.80	32.40	24.20
L-Threonine	10.40	12.60	11.20	13.90	7.60	23.90	6.30	9.90	11.00	9.40	8.30	13.10	15.20	20.90	25.20	21.90
L-Phenylalanine	9.20	9.90	12.10	12.40	15.50	25.30	8.90	9.40	11.00	12.40	9.80	12.60	14.90	21.4	35.40	24.00
L-Asparticacid	23.10	24.50	24.40	29.60	32.00	45.30	12.70	25.70	29.70	29.40	27.60	32.30	35.00	44.90	54.60	45.90
Hidroxiprolina	1.20	1.70	1.40	2.00	1.50	2.40	3.40	1.70	1.70	2.00	1.40	2.20	2.20	1.60	2.70	2.00
L-Cysteine	1.40	1.70	1.40	2.20	1.70	2.40	2.80	1.70	1.60	1.50	1.40	1.50	2.00	2.90	3.00	2.10
L-Glutamicacid	25.30	25.20	29.50	27.60	25.60	40.90	16.60	29.80	30.60	29.90	26.70	26.40	30.30	35.40	48.70	39.10
L-Asparagine	0.50	0.70	0.60	0.50	0.50	0.60	1.10	0.50	0.40	0.50	0.30	0.50	0.50	0.50	1.00	0.40
L-Lysine	10.40	8.40	11.60	14.40	11.30	19.20	7.10	7.20	7.60	8.20	13.10	15.80	11.40	21.70	25.20	19.00
L-Glutamine	1.00	1.20	3.40	0.50	0.70	1.60	0.90	nd	1.00	1.50	nd	nd	0.50	0.30	1.50	0.90
L-Cystine	1.70	2.20	2.20	2.50	2.50	3.30	4.80	2.50	2.30	2.50	1.70	2.50	2.70	2.20	4.40	2.50
L-Arginine	34.00	36.60	73.00	36.00	21.10	57.60	30.10	85.00	86.70	94.40	72.40	57.90	60.10	46.30	93.60	56.60
L-Histidine	1.20	1.50	2.10	1.70	1.70	2.30	0.70	1.50	1.30	2.00	1.60	3.00	2.00	1.70	4.20	2.10
L-Tyrosine	5.50	5.20	7.50	6.20	7.60	16.80	5.40	6.40	7.00	8.60	6.90	6.70	9.90	15.00	20.80	15.40
L-Tryptophan	2.90	4.00	3.40	4.00	3.90	3.40	10.10	3.90	3.40	4.00	2.80	3.90	4.00	2.80	4.00	3.40
ΣEssential AA	87.50 ^k^	87.10 ^l^	101.10 ^i^	113.20 ^g^	113.90 ^f^	167.50 ^c^	85.90 ^m^	79.10 ^p^	84.60 ^n^	91.90 ^j^	84.20 ^o^	110.30 ^h^	117.60 ^e^	151.20 ^d^	213.00 ^a^	170.50 ^b^
ΣNon-essential AA	173.60 ^o^	175.80 ^n^	226.00 ^j^	217.00 ^l^	193.70 ^m^	316.90 ^b^	144.40 ^p^	236.70 ^i^	246.70 ^g^	256.00 ^f^	224.40 ^k^	240.90 ^h^	256.50 ^e^	276.70 ^d^	410.60 ^a^	312.80 ^c^

Essential AA—essential amino acids; Non-essential AA—non-essential amino acids; nd—not detected. Different letters on the same line indicate differences by Tukey’s test (*p* < 0.05).

**Table 3:** Sterols profile (mg kg^−1^) for *Scenedesmus obliquus* in different photo-cycles.

Compounds	Long-Term Photo-Cycle						Frequency Photo-Cycle						Short Photo-Cycle			
	24:0	22:2	20:4	18:6	12:12	10:14	2 t/d	4 t/d	8 t/d	12 t/d	24 t/d	48 t/d	0.91:0.09	0.83:0.17	0.75:0.25	0.50:0.50
Squalene	6.77	4.12	1.59	2.79	3.30	2.46	5.66	7.89	5.02	3.92	2.32	1.01	3.93	10.70	1.64	6.40
Tocopherol	nd	12.76	nd	nd	nd	nd	36.95	22.96	21.84	28.68	nd	nd	nd	nd	nd	nd
Ergosterol	32.86	10.26	14.43	27.70	14.81	23.74	142.52	79.49	92.15	28.15	15.92	6.80	33.53	79.78	43.32	66.13
Stigmasterol	83.56	32.99	35.35	118.33	49.99	66.18	450.16	265.91	239.90	115.68	54.70	22.06	112.74	276.55	130.98	196.31
β-sitosterol	nd	nd	nd	37.05	nd	nd	99.98	68.62	63.04	37.03	30.37	nd	36.60	52.28	36.43	40.35
∑Total sterol	123.19 ^j^	60.13 ^n^	51.37 ^o^	185.87 ^i^	68.10 ^m^	92.38 ^l^	735.2 ^a^	444.87 ^b^	421.95 ^c^	213.46 ^f^	103.31 ^k^	29.87 ^p^	186.80 ^h^	419.31 ^d^	212.37 ^g^	309.19 ^e^

nd—not detected. Different letters on the same line indicate differences by Tukey’s test (*p* < 0.05).
